# Supplementary material for: Predicting visual function by interpreting a neuronal wiring diagram
Source: Nature. 2024 Oct 2;634(8032):113–23. doi: 10.1038/s41586-024-07953-5 (PMC11446822; doi:10.1038/s41586-024-07953-5)
Supplement: Supplementary file 6 — CRF and ERF predictions for individual TmY4 and TmY9 cells. Analogous to Supplementary Data 3, but for TmY target types. Shown are the top four monosynaptic pathways, the strongest pathway passing through each of the top ten intermediary types (ranking from Extended Data Fig. 7), and the trisynaptic pathway Tm1–TmY–Dm3–TmY (see the section entitled Prediction of spatial normalization). [file 41586_2024_7953_MOESM6_ESM.zip › DataS4/TmY9q/720575940630501246.pdf]

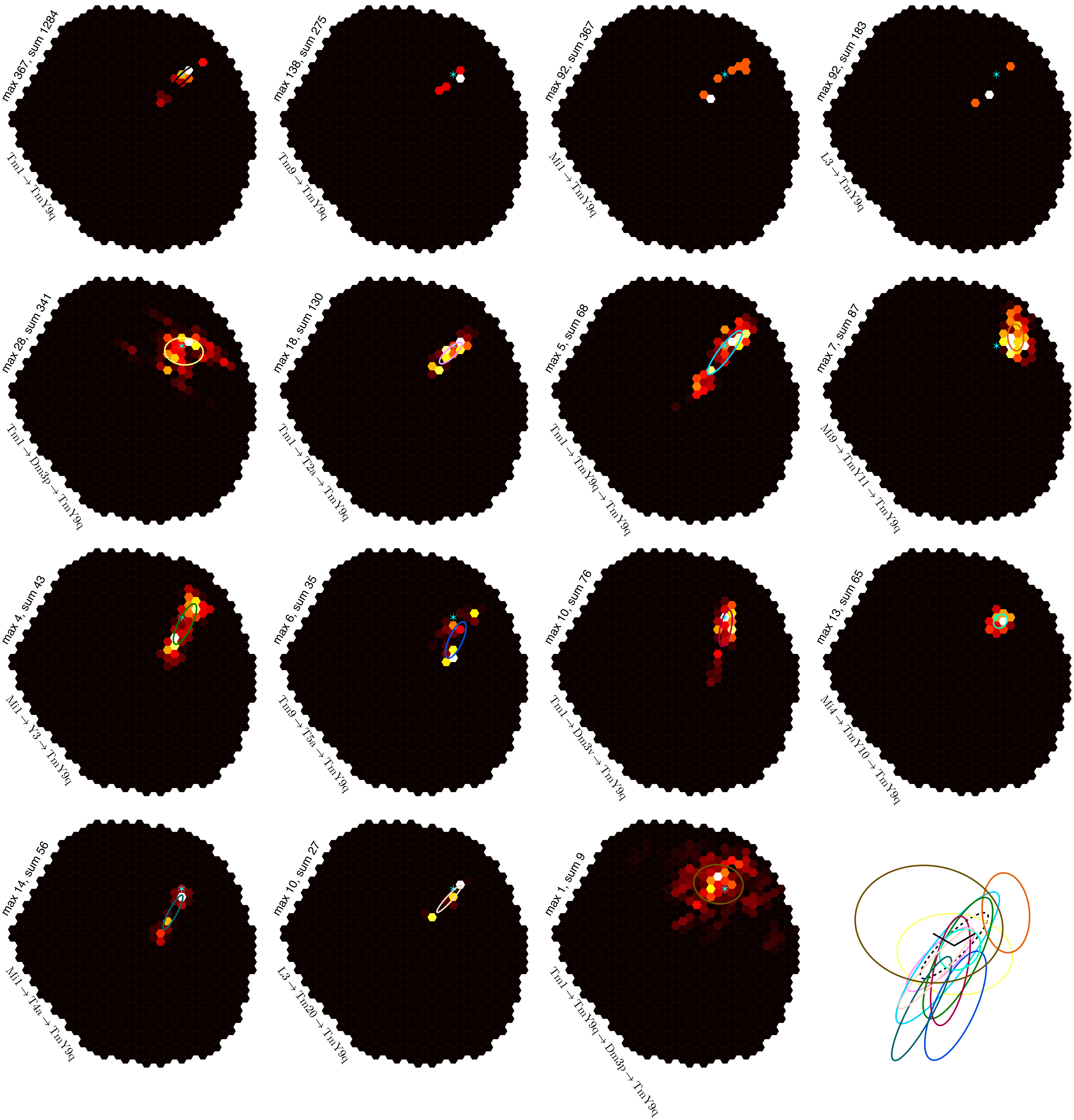
max 367, sum 1284
Tm1 → TmY9q
max 138, sum 275
Tm9 → TmY9q
max 92, sum 367
Mi1 → TmY9q
max 92, sum 183
L3 → TmY9q
max 28, sum 341
Tm1 → Dm3p → TmY9q
max 18, sum 130
Tm1 → T2a → TmY9q
max 5, sum 68
Tm1 → TmY9q → TmY9q
max 7, sum 87
Mi9 → TmY11 → TmY9q
max 4, sum 43
Mi1 → Y3 → TmY9q
max 6, sum 35
Tm9 → T5a → TmY9q
max 10, sum 76
Tm1 → Dm3v → TmY9q
max 13, sum 65
Mi4 → TmY10 → TmY9q
max 14, sum 56
Mi1 → T4a → TmY9q
max 10, sum 27
L3 → Tm20 → TmY9q
max 1, sum 9
Tm1 → TmY9q → Dm3p → TmY9q
